# Collection kystique retro péritonéal révélant un énorme urinoma

**DOI:** 10.11604/pamj.2016.23.7.8638

**Published:** 2016-01-20

**Authors:** Anouar El Ghazoui, Amine Slaoui

**Affiliations:** 1 Service d'Urologie B, CHU Ibn Sina, Rabat, Maroc

**Keywords:** Collection, retro péritonéale, kystique, urinoma, Retroperitoneal, cystic, collection, urinoma

## Image en médecine

Madame RF âgée de 50 ans, diabétique sous insuline mal suivie compliquée d'une neuropathie optique au stade de pré-cécité, avec une artériopathie oblitérante du membre inferieure. La patiente consultait pour les lombalgies. A l'examen clinique on retrouve une patiente apyrétique, présentant un contact lombaire. La bandelette urinaire était négative. Le bilan biologique était correct, la sérologie hydatique était négative. Sur le plan radiologique, l’échographie abdominale mettait en évidence une énorme masse liquidienne rétro-péritonéale gauche refoulant le rein gauche vers l'arrière. L'Uro-scanner retrouvait une masse kystique de 18 cm, rétro péritonéale et occupant la quasi-totalité de cet espace. Sur les coupes coronales, elle refoulait vers l'avant le péritoine. Par ailleurs, l'absence de dilatation des voies excrétrices nous permet d’évoquer les hypothèses suivantes: une rupture de fornix, une duplicité rénale ou un lymphangiome kystique. Enfin l'Uretero-pyelographie rétrograde a permis de trancher pour un urinome, car elle a mis en exergue l'extravasation du PC. En ce qui concerne la pris en charge, une sonde JJ a été laissée en place jusqu’à assèchement de la collection

**Figure 1:**
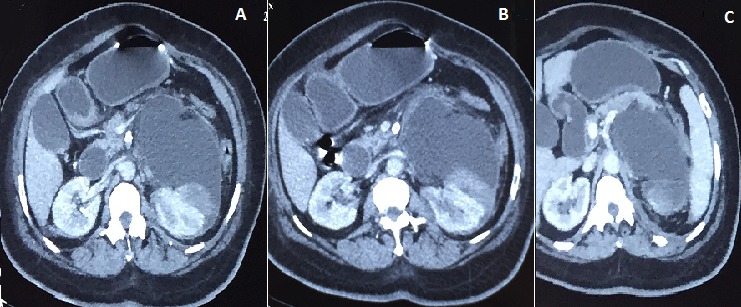
(A) masse kystique retro-péritonéale de 18 cm; (B) retro -peritonealcystic mass of 18 cm

